# Clinical Characteristics and Outcome of Patients With Hemorrhagic Transformation After Intravenous Thrombolysis in the WAKE-UP Trial

**DOI:** 10.3389/fneur.2020.00957

**Published:** 2020-08-28

**Authors:** Märit Jensen, Eckhard Schlemm, Bastian Cheng, Iris Lettow, Fanny Quandt, Florent Boutitie, Martin Ebinger, Matthias Endres, Jochen B. Fiebach, Jens Fiehler, Ivana Galinovic, Vincent Thijs, Robin Lemmens, Keith W. Muir, Norbert Nighoghossian, Salvador Pedraza, Claus Z. Simonsen, Christian Gerloff, Götz Thomalla

**Affiliations:** ^1^Klinik und Poliklinik für Neurologie, Kopf- und Neurozentrum, University Medical Center Hamburg-Eppendorf, Hamburg, Germany; ^2^Hospices Civils de Lyon, Service de Biostatistique, Lyon, France; ^3^Université Lyon 1, Villeurbanne, France; ^4^CNRS, UMR 5558, Laboratoire de Biométrie et Biologie Evolutive, Equipe Biostatistique-Santé, Villeurbanne, France; ^5^Centrum für Schlaganfallforschung Berlin (CSB), Charité—Universitätsmedizin Berlin, Berlin, Germany; ^6^Neurologie, Medical Park Berlin Humboldtmühle, Berlin, Germany; ^7^Klinik und Hochschulambulanz für Neurologie, Charité-Universitätsmedizin Berlin, Berlin, Germany; ^8^German Center for Neurodegenerative Disease (DZNE), Partner Site Berlin, Berlin, Germany; ^9^German Center for Cardiovascular Research (DZHK), Partner Site Berlin, Berlin, Germany; ^10^Department of Diagnostic and Interventional Neuroradiology, University Medical Center Hamburg-Eppendorf, Hamburg, Germany; ^11^Florey Institute of Neuroscience and Mental Health, University of Melbourne, Heidelberg, VIC, Australia; ^12^Austin Health, Department of Neurology, Heidelberg, VIC, Australia; ^13^Department of Neurology, University Hospitals Leuven, Leuven, Belgium; ^14^Department of Neurosciences, Experimental Neurology, KU Leuven—University of Leuven, Leuven, Belgium; ^15^VIB, Laboratory of Neurobiology, Center for Brain & Disease Research, Leuven, Belgium; ^16^Institute of Neuroscience and Psychology, University of Glasgow, Glasgow, United Kingdom; ^17^Department of Stroke Medicine, Université Claude Bernard Lyon 1, Hospices Civils de Lyon, Lyon, France; ^18^Department of Radiology, Institut de Diagnostic per la Image (IDI), Hospital Dr. Josep Trueta, Institut d'Investigació Biomèdica de Girona (IDIBGI), Girona, Spain; ^19^Department of Neurology, Aarhus University Hospital, Aarhus, Denmark

**Keywords:** ischemic stroke, WAKE-UP, thrombolysis, intracerebral hemorrhage, hemorrhagic transformation

## Abstract

**Background:** Hemorrhagic transformation (HT) is an important complication of intravenous thrombolysis with alteplase. HT can show a wide range from petechiae to parenchymal hematoma with mass effect with varying clinical impact. We studied clinical and imaging characteristics of patients with HT and evaluated whether different types of HT are associated with functional outcome.

**Methods:** We performed a *post-hoc* analysis of WAKE-UP, a multicenter, randomized, placebo-controlled trial of MRI-guided intravenous alteplase in unknown onset stroke. HT was assessed on follow-up MRI or CT and diagnosed as hemorrhagic infarction type 1 and type 2 (HI1 and HI2, combined as HI), and parenchymal hemorrhage type 1 and type 2 (PH1 and PH2, combined as PH). Severity of stroke symptoms was assessed using the National Institutes of Health Stroke Scale (NIHSS) at baseline. Stroke lesion volume was measured on baseline diffusion weighted imaging (DWI). Primary endpoint was a favorable outcome defined as a modified Rankin Scale score 0–1 at 90 days.

**Results:** Of 483 patients included in the analysis, 95 (19.7%) showed HI and 21 (4.4%) had PH. Multiple logistic regression analysis identified treatment with alteplase (OR, 2.08 [95% CI, 1.28–3.40]), baseline NIHSS score (OR, 1.11 [95% CI, 1.05–1.17]), DWI lesion volume (OR, 1.03 [95% CI, 1.01–1.05]), baseline glucose levels (OR, 1.01 [95% CI, 1.00–1.01]) and atrial fibrillation (OR, 3.02 [95% CI, 1.57–5.80]) as predictors of any HT. The same parameters predicted HI. Predictors of PH were baseline NIHSS score (OR, 1.11 [95% CI, 1.01–1.22]) and as a trend treatment with alteplase (OR, 2.40 [95% CI, 0.93–6.96]). PH was associated with lower odds of favorable outcome (OR 0.25, 95% [CI 0.05–0.86]), while HI was not.

**Conclusion:** Our results indicate that HI is associated with stroke severity, cardiovascular risk factors and thrombolysis. PH is a rare complication, more frequent in severe stroke and with thrombolysis. In contrast to HI, PH is associated with worse functional outcome. The impact of HT after MRI-guided intravenous alteplase for unknown onset stroke on clinical outcome is similar as in the trials of stroke thrombolysis within a known early time-window.

## Introduction

Hemorrhagic transformation (HT) represents an important complication of intravenous thrombolysis with alteplase for acute ischemic stroke. However, HT of ischemic stroke can show a wide range from small petechiae with no clinical impact to massive parenchymal hematoma with space-occupying effect associated with neurological deterioration. The following four subtypes of HT have been distinguished radiologically: hemorrhagic infarction type 1 (HI1; scattered small petechiae, no mass effect), hemorrhagic infarction type 2 (HI2; confluent petechiae, no mass effect), parenchymal hemorrhage type 1 (PH1; hematoma within infarcted tissue, occupying < 30%, no substantive mass effect) and parenchymal hemorrhage type 2 (PH2; hematoma occupying 30% or more of the infarcted tissue, with obvious mass effect) (1). In addition, intracerebral hemorrhage outside infarcted brain tissue or intracranial-extracerebral hemorrhage is considered a separate category, including subarachnoidal hemorrhage and subdural hematoma. The clinical significance of different types of HT after thrombolysis is a matter of debate (2). While there is no doubt that massive HT, meeting criteria of PH2, is likely to be associated with clinical worsening, mere hemorrhagic infarction (HI) may also be understood as a marker of successful recanalization into partially ischemic damage with no adverse clinical effect (3, 4). Previous work has suggested a different pathogenesis for HI and PH. While HI might be a clinically irrelevant epiphenomenon of ischemic damage and reperfusion, PH appears to be related to biological effects of alteplase and other pre-existing pathologic conditions and also carries the potential of clinical deterioration (4). At the same time, it is still uncertain how—if at all—patients at high risk of severe intracerebral hemorrhage after thrombolysis can be identified beforehand based on clinical or imaging characteristics. In the present study, our first objective was to identify possible clinical and imaging parameters that predict HT after acute ischemic stroke. Second, we aimed to study the functional outcome of patients with different types of HT to get further insights into the clinical significance of HT after intravenous thrombolysis.

## Methods

### Study Design

In this exploratory *post-hoc* analysis of the WAKE-UP trial, we reviewed patients for intracerebral hemorrhage on follow-up imaging 22–36 h after stroke. WAKE-UP was a multicenter, randomized, double-blind, placebo-controlled clinical trial to study the efficacy and safety of intravenous thrombolysis with alteplase in patients with an acute stroke of unknown onset time, guided by MRI. Inclusion criteria comprised the mismatch between an acute ischemic lesion visible on diffusion-weighted imaging (DWI) but with no corresponding marked parenchymal hyperintensity on fluid-attenuated inversion recovery (FLAIR) as a surrogate marker of lesion age, indicating that the stroke had most likely occurred within 4.5 h (5). Patients or their legal representatives provided written informed consent according to national and local regulations. There was an exception from explicit informed consent in emergency circumstances in some countries. For each study site, the competent authorities and the corresponding ethics committee approved the trial. The detailed trial protocol has been published together with its main results (5). The trial was registered at ClinicalTrials.gov number (NCT01525290) and EudraCT (2011-005906-32).

In this analysis, we examined demographic characteristics, medical history, clinical and imaging data at baseline and follow-up, including final follow-up at 90 days after stroke. HT was assessed on MRI or, if MRI was not feasible, on CT 22–36 h after randomization and categorized into established radiologic subtypes HI1, HI2, PH1, PH2, and other hemorrhages (1). Image reading was performed by the central image reading board consisting of experienced neuroradiologists. For statistical analysis, we collapsed the subtypes of HT into two categories HI (HI1 and HI2), and PH (PH1 and PH2). Other subtypes of hemorrhages outside the infarcted brain tissue or intracranial-extracerebral hemorrhage were disregarded in the current analysis. DWI lesions were segmented and quantified on apparent diffusion coefficient (ADC) maps calculated from DWI using a semi-automated procedure based on an upper ADC threshold of 620 mm^2^/s.

### Outcome Measures and Endpoints

Clinical outcome was assessed at 90 days after stroke. The primary endpoint was favorable outcome defined as a score of 0–1 on the modified Rankin Scale (mRS). Secondary endpoint in this analysis comprised the ordinal analysis of the mRS (“shift analysis”).

### Statistical Analysis

Baseline characteristics were compared between patients with and without hemorrhage using Chi-square test, *t*-tests, ANOVA or non-parametric Kruskall-Wallis test as appropriate. In addition, multiple logistic regression analysis was used to assess the association between HT and baseline clinical and imaging characteristics in a joint model. To investigate the association between HT and functional outcome, we fitted three separate unconditional logistic regression models to associate the log odds of achieving a mRS score of 0–1 with the occurrence of any HT, HI, or PH. All models were adjusted for the stratification parameters age and NIHSS score, treatment group, and parameters that were predictive of any HT, HI, or PH in multivariate analysis (i.e., baseline NIHSS score, baseline glucose levels, atrial fibrillation (AF), and DWI lesion volume). Odds ratios were tested against the null hypothesis of no association using *t-*tests and presented with 95% confidence intervals obtained from profiling the likelihood function. Associations between HT and a shift in the distribution of mRS scores were assessed by fitting ordinal logistic regression models under the proportional odds assumptions with the same covariates as above. All tests were carried out with a two-sided significance level of 5% without correction for multiple comparisons.

## Results

### Patient Characteristics

**Figure 1:**
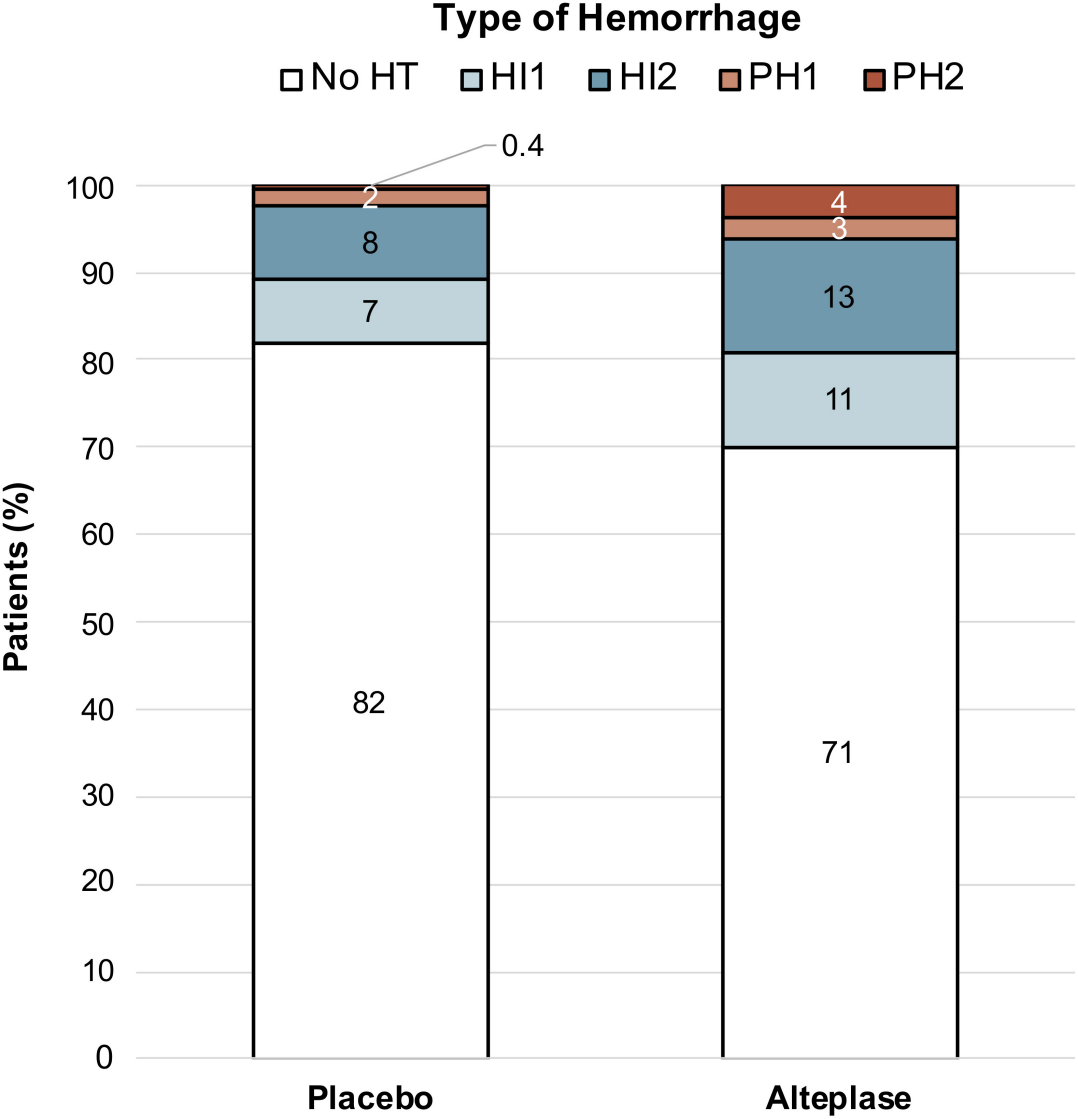
Distribution of Hemorrhages in relation to the treatment group. Distribution of Hemorrhage in relation to the treatment groups shows that any hemorrhage was more frequent in patients treated with alteplase. While HI occurred in both treatment groups, PH was mainly seen in patients treated with alteplase. ICH, intracerebral hemorrhage; HI1, hemorrhagic infarction type 1; HI2, hemorrhagic infarction type 2; PH1, parenchymal hemorrhage type 1; PH2, parenchymal hemorrhage type 2.

Of 503 patients randomized in WAKE-UP, follow-up imaging and data on the primary endpoint was available for 486 patients. Follow-up imaging was performed by MRI in 457 (94%) patients and by CT in 29 patients (6%). Three patients with hemorrhage outside the infarcted brain tissue were excluded from further analysis. HT, either HI or PH, was present in 116 (24%) patients. We observed HI1 in 44 (9.1%), HI2 in 51 (10.6%), PH1 in 11 (2.3%), and PH2 in 10 (2.1%) patients. Figure 1 illustrates the distribution of HT type in relation to the treatment group. Subgroup analysis revealed that PH2 was more frequent with alteplase as compared to placebo (3.7 vs. 0.41%, *p* = 0.01), while HI1 (10.8 vs. 7.45%, *p* = 0.26), HI2 (12.9 vs. 8.3%, *p* = 0.14), and PH1 (2.5 vs. 2.1%, *p* = 0.99) did not show significant differences between treatment groups.

Clinical and imaging characteristics of patients with and without HT are shown in Table 1. Treatment with alteplase was more prevalent in patients with HT (62 vs. 46%, *p* = 0.004). Those with hemorrhage were more severely affected, with a higher median NIHSS score on admission (9 vs. 5, *p* < 0.001), and more often had cardiovascular comorbidities including AF (25 vs. 9%, *p* < 0.001) and diabetes mellitus (26 vs. 13%, *p* = 0.002). Accordingly, medication with antidiabetics was more frequent (23 vs. 11%, *p* = 0.001), and glucose levels on admission were higher in patients with HT (median 124 vs. 113 mg/dL, *p* < 0.001). DWI lesion volume at baseline was significantly higher (median 7.33 vs. 1.53 mL, *p* < 0.001) in patients with hemorrhage.

**Table 1:** Baseline characteristics of patients with and without HT.

	**Patients, No. (%)**					
**Variable**	**No HT (*n =* 367)**	**Any HT (*n =* 116)**	**Group comparison No HT vs. any HT *p-*value**	**HI (*n =* 95)**	**PH (*n =* 21)**	**Group comparison No HT vs. HI vs. PH *p-*value**
Age, mean (SD), y	64.8 (11.7)	66.4 (10.9)	0.19	66.4 (11.3)	66.4 (9.38)	0.45
Female	128 (34.9)	44 (37.9)	0.63	36 (37.9)	8 (38.1)	0.84
Medical history or risk factors						
Arterial hypertension	187 (51.2)	67 (58.3)	0.23	58 (61.7)	9 (42.9)	0.12
Diabetes mellitus	48 (13.2)	30 (26.3)	0.002	28 (30.1)	2 (9.5)	0.001
Hypercholesterolemia	128 (36.4)	46 (42.2)	0.32	43 (48.9)	3 (14.3)	0.007
Atrial fibrillation	31 (8.6)	28 (24.6)	<0.001	23 (24.5)	5 (25)	<0.001
History of ischemic stroke	49 (13.4)	17 (14.7)	0.85	15 (15.8)	2 (9.5)	0.79
Medication classes						
Antiplatelets	118 (32.2)	40 (34.5)	0.72	34 (35.8)	6 (28.6)	0.73
Statins	106 (28.9)	43 (37.1)	0.12	39 (41.1)	4 (19)	0.04
Antihypertensives	173 (47.1)	62 (53.4)	0.28	52 (54.7)	10 (47.6)	0.42
Antidiabetics	40 (10.9)	27 (23.3)	0.001	26 (27.4)	1 (4.8)	<0.001
Laboratory parameters						
Platelet count, median (IQR), 10^3^/μL	230 (191–273)	222 (189–270)	0.67	226 (188-281)	200 (195–232)	0.34
Serum glucose, median (IQR), mg/dL	113 (101–133)	124 (107–164)	<0.001	124 (107–167)	124 (110–146)	0.001
National Institute of Health Stroke Scale score, median (IQR)	5 (3–8)	9 (6–15)	<0.001	9 (5–15)	11 (6–12)	<0.001
Diffusion-weighted imaging lesion volume at baseline, median (IQR), mL	1.53 (0.61–5.68)	7.33 (3.16–23)	<0.001	7.02 (3.12–22.7)	10.3 (5.1–23.3)	<0.001
Time from last-seen-well to treatment initiation, median (IQR), min	605 (485–714)	660 (520–751)	0.044	658 (537–739)	690 (455–815)	0.12
Time from symptom recognition to treatment initiation, median (IQR), min	186 (155–230)	192 (144–236)	0.72	192 (146–234)	175 (133–240)	0.72
Treatment with Alteplase	169 (46)	72 (62.1)	0.004	57 (60)	15 (71.4)	0.007

*HT, hemorrhagic transformation; HI, hemorrhagic infarction; PH, parenchymal hemorrhage; SD, standard deviation; IQR, interquartile range*.

### Clinical and Imaging Predictors of Hemorrhagic Transformation

For analysis, data from 29 patients were excluded due to missing data (i.e., 1 patient without information on glucose level and AF, 11 patients without information on glucose levels, 7 patients without information on AF, and 10 patients in whom quantification of DWI lesion volume was not possible). Among all randomized patients, the following parameters were associated with any HT on follow-up in multiple regression analysis (see Table 2): treatment with alteplase (OR, 2.08 [95% CI, 1.28–3.40]), baseline NIHSS score (OR, 1.11 [95% CI, 1.05–1.17]), DWI lesion volume (OR, 1.03 [95% CI, 1.01–1.05]), serum glucose levels (OR, 1.01 [95% CI, 1.00–1.01]), and AF (OR, 3.02 [95% CI, 1.57–5.80]). HI was predicted by treatment with alteplase (OR, 1.73 [95% CI, 1.05–2.89]), baseline NIHSS score (OR, 1.08 [95% CI, 1.02–1.14]), DWI lesion volume (OR, 1.03 [95% CI, 1.01–1.05]), serum glucose (OR, 1.01 [95% CI, 1.00–1.01]), and AF (OR, 2.58 [95% CI, 1.32–4.97]). NIHSS score on admission predicted the occurrence of PH (OR, 1.11 [95% CI, 1.01–1.22]), and there was a trend for an association of alteplase treatment with PH (OR, 2.40 [95% CI, 0.93–6.96]).

**Table 2:** Clinical and imaging predictors of any HT, HI, and PH.

	***p*-value**	OR^*^ (95% CI)
**Predictors of any HT**		
National Institute of Health Stroke Scale score	<0.001	1.11 (1.05–1.17)
Baseline blood glucose	0.004	1.01 (1.00–1.01)
Atrial fibrillation	<0.001	3.02 (1.57–5.80)
Diffusion-weighted imaging lesion volume at baseline, mL	0.005	1.03 (1.01–1.05)
Age, y	0.32	1.01 (0.99–1.04)
Treatment with Alteplase	0.003	2.08 (1.28–3.40)
**Predictors of HI**		
National Institute of Health Stroke Scale score	0.005	1.08 (1.02–1.14)
Baseline blood glucose	0.003	1.01 (1.00–1.01)
Atrial fibrillation	0.005	2.58 (1.32–4.97)
Diffusion-weighted imaging lesion volume at baseline, mL	0.006	1.03 (1.01–1.05)
Age, y	0.42	1.01 (0.99–1.04)
Treatment with Alteplase	0.033	1.73 (1.05–2.89)
**Predictors of PH**		
National Institute of Health Stroke Scale score	0.027	1.11 (1.01–1.22)
Baseline blood glucose	0.90	1.00 (0.99–1.01)
Atrial fibrillation	0.26	1.90 (0.57–5.43)
Diffusion-weighted imaging lesion volume at baseline, mL	0.80	1.00 (0.97–1.04)
Age, y	0.58	1.01 (0.97–1.07)
Treatment with Alteplase	0.08	2.40 (0.93–6.96)

*HT, hemorrhagic transformation; HI, hemorrhagic infarction; PH, parenchymal hemorrhage; OR, odds ratio; CI, confidence interval*.

* *multiple logistic regression analysis*.

### Influence of Intracerebral Hemorrhage on Stroke Outcome

**Figure 2:**
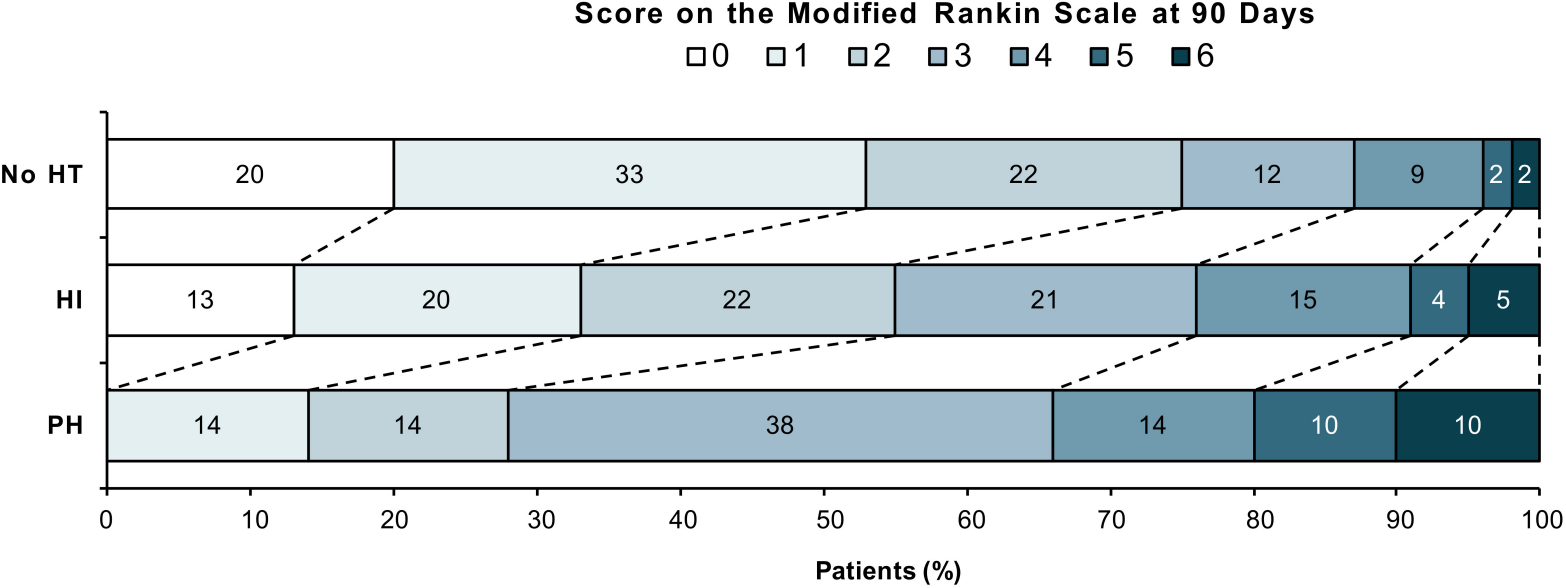
Distribution of Modified Rankin Scale scores at 90 days after stroke by hemorrhage groups. Modified Rankin Scale scores range from 0 to 6 (0, no symptoms; 1, no clinically significant disability; 2, slight disability; 3, moderate disability; 4, moderately severe disability; 5, severe disability; and 6, death). HT, hemorrhagic transformation; HI, hemorrhagic infarction; PH, parenchymal hemorrhage.

Figure 2 shows the distribution of modified Rankin Scale scores at 90 days after stroke by hemorrhage group. Multivariate analysis adjusted for age, baseline NIHSS, treatment group, AF, serum glucose and DWI lesion volume revealed an independent association of PH with lower odds of favorable outcome. Favorable outcome was observed in 3 of 21 patients (14.3%) in the PH group and in 227 of 462 patients (49.1%) in the group without PH [OR, 0.25 [95% CI, 0.05–0.86]; see Table 3]. In ordinal analysis of the mRS at 90 days, PH was associated with a shift toward worse functional outcomes (OR, 0.39 [95% CI, 0.17–0.89]). Neither any HT nor HI showed a significant association with outcome.

**Table 3:** Association of any HT, HI, and PH with favorable functional outcome.

	**Type of hemorrhage**					
	**Any HT (HI and PH)**		**HI**		**PH**	
**Assessment Variable at 90 days**	OR^*^ (95% CI)	***p*-value**	OR^*^ (95% CI)	***p*-value**	OR^*^ (95% CI)	***p*-value**
Primary end point (Modified Rankin Scale score 0–1)	0.64 (0.37–1.09)	0.10	0.83 (0.47–1.46)	0.52	0.25 (0.05–0.86)	0.043
Secondary end point (“Shift analysis”)	0.69 (0.46–1.06)	0.09	0.88 (0.57–1.36)	0.56	0.39 (0.17–0.89)	0.026

* *Multiple logistic regression analysis including age > 60, baseline NIHSS score > 10, DWI lesion volume, treatment with alteplase, baseline glucose, and atrial fibrillation. HT, hemorrhagic transformation; HI, hemorrhagic infarction; PH, parenchymal hemorrhage; OR, odds ratio; CI, confidence interval*.

## Discussion

In this *post-hoc* analysis of HT after intravenous thrombolysis for acute ischemic stroke in the WAKE-UP trial we demonstrate that: (1) HI, although slightly more prevalent in alteplase-treated patients, may occur as part of natural history of ischemic stroke with or without thrombolysis, while severe HT was more frequently seen in the context of alteplase treatment; (2) treatment with alteplase, baseline NIHSS Score, DWI lesion volume, serum glucose levels, and AF are predictors of HT and HI, and (3) PH but not HI was associated with worse functional outcome after correction for baseline predictors.

In our cohort, HT occurred in 18.2% of patients with placebo and in 29.9% with alteplase. The observed rate of hemorrhage was slightly lower as compared to a pooled analysis of previous clinical trials of stroke thrombolysis, in which radiographic evidence of HT occurred in 24.2% of placebo-treated patients and 32.5% of alteplase-treated patients (6, 7). However, the distribution between both groups was similar. The lower rate of hemorrhage is also remarkable, as we used MRI for follow-up imaging in the vast majority (>90%) of patients, and MRI is known to be more sensitive to HT than CT (8), which was the main follow-up imaging modality in the pooled stroke thrombolysis trials. Amongst other factors, the overall lower rate of HT in our population may be attributable to the trial design, which led to the exclusion of very elderly patients (>80 years of age) as age was associated with HT in other studies (9–11). The fact that our population was less severely affected with a median NIHSS of 6 as compared to 11 in previous pooled stroke thrombolysis trials may also have contributed to overall lower rates of HT.

It has been suggested that the underlying mechanisms of HI and PH differ from each other (12). Our results showed that HI occurred in 15.7% of placebo-treated patients and 23.7% of alteplase-treated patients. In contrast, PH is uncommon and was found in only 2.5% of placebo-treated patients, but in 6.2% of alteplase-treated patients. Especially PH2 (0.4 vs. 3.7%, *p* = 0.01) was associated with intravenous thrombolysis. These findings are in line with previous studies, that showed that HI is also encountered without the use of thrombolytic agents and thus occurs as a part of natural history of ischemic stroke, while severe PH appears mainly attributable to biological effects of treatment with alteplase (12).

However, the coagulopathy induced by alteplase is not the only determinant of HT occurring after ischemic stroke. We additionally identified NIHSS score and DWI lesion volume on admission, blood glucose levels and AF as independent predictors of HT. The same risk factors were observed for HI, which accounts for the majority of HTs (i.e., 82%). These results are in line with previous findings. DWI lesion volume was associated with the occurrence of HT/HI (13). Higher NIHSS score values, which were also associated with HT, also reflect more severe strokes and larger infarcts (4). The association of AF with HT that we observed in our analysis has also been reported in previous studies (14–16). AF is associated with higher volumes of more severe baseline hypoperfusion leading to greater infarct growth (17). The more severe ischemia with this type of stroke has been postulated to damage blood vessel integrity, resulting in increased HT, especially with reperfusion (18). Elevated glucose levels are usually considered to be a risk factor for HT, especially in the setting of thrombolysis (19). Accordingly, in our study patients with HT had higher baseline glucose levels. Experimental studies have suggested that several pathological mechanisms, e.g., increased activity of matrix metalloproteinases and enhanced apoptosis of smooth muscle cells, might result in diffuse damage of the microvasculature and thus increase infarct size and risk of HT (20).

Although the absolute difference between the percentage of patients treated with alteplase in the PH and no HT group was high (71 vs. 46%), multiple regression analysis failed to demonstrate a significant association of alteplase treatment with PH (*p* = 0.08). In our study, solely NIHSS score on admission was significantly associated with PH (*p* = 0.027). The reviewed literature additionally identifies large lesions attributable to cardioembolism, hyperglycemia, extent of parenchymal hypoattenuation on baseline CT scan, a history of congestive heart failure, increasing age, and baseline systolic blood pressure as predictors of PH (9, 21, 22). We assume that the low number of patients with PH in our study limits the statistical power to detect possible associations of risk factors with PH.

Previous studies have suggested that most types of HT in acute stroke do not have a relevant effect on the clinical outcome in the majority of cases. On the contrary it has been suggested that mild to moderate HT represents a marker of successful treatment and vascular recanalization (4, 23, 24). In support of this notion, in our analysis HI was not associated with poor outcome at 3 months. In contrast and in agreement with previous studies, PH was associated with worse functional outcome (2, 9).

There are limitations to our study. Due to the observational design of the analysis of hemorrhage, we cannot claim a causal relationship between hemorrhage and functional outcome. In addition, as study inclusion and exclusion criteria entailed a selected and relatively young sample of stroke patients, generalizability of our results is limited. Finally, the small number of patients with severe intracerebral hemorrhagic complications limits the statistical power of our analysis.

## Conclusion

HT is a frequent observation in acute ischemic stroke and more frequent with intravenous thrombolysis as compared to placebo. Our results support the hypothesis that HI pathogenesis may be related to stroke severity, cardiovascular risk factors and thrombolysis but does not negatively influence stroke outcome. PH is a rare complication, more frequent in severe stroke and after treatment with alteplase, and is associated with worse functional outcome. The impact of HT in the WAKE-UP trial of MRI-guided intravenous alteplase for unknown onset stroke on clinical outcome is similar as in the trials of stroke thrombolysis within a known early time-window.

## Data Availability Statement

The raw data supporting the conclusions of this article will be made available by the authors, without undue reservation.

## Ethics Statement

The studies involving human participants were reviewed and approved by the ethics committee of the Hamburg chamber of physicians, Weidestr. 122b, 22083 Hamburg, Germany was the primary ethics committee that approved the trial (PVN3857). For each study site, the competent authorities and the corresponding ethics committee approved the trial. The patients/participants provided their written informed consent to participate in this study.

## Author Contributions

MJ and GT developed the study protocol, interpreted the data, and drafted the manuscript. ES and FB provided support with statistical analysis. BC, MEb, MEn, JBF, JF, IG, VT, RL, KM, NN, SP, CS, CG, and GT collected data. ES, BC, IL, FQ, FB, MEb, MEn, JBF, JF, IG, VT, RL, KM, NN, SP, CS, and CG interpreted the data and edited the manuscript. All authors contributed to the article and approved the submitted version.

## Conflict of Interest

FB reports grants from University Medical Center Hamburg-Eppendorf during the conduct of the study. BC reports grants from European Union 7th Framework Program during the conduct of the study. MEb reports grants from European Union 7th Framework Program during the conduct of the study. MEn reports grant support from Bayer, the German Research Foundation (DFG), the German Federal Ministry of Education and Research (BMBF), the German Center for Neurodegenerative Diseases (DZNE), the German Center for Cardiovascular Research (DZHK), the European Union, Corona Foundation, and Fondation Leducq; and fees paid to the Charité from Bayer, Boehringer Ingelheim, Bristol-Myers Squibb, Pfizer, Daiichi Sankyo, Amgen, GlaxoSmithKline, Sanofi, Covidien, Novartis, all outside the submitted work. JBF reports grants from European Union 7th Framework Program during the conduct of the study and personal fees from Bioclinica, Artemida, Cerevast, Brainomix, BMS, Merck, Eisai, Biogen, Guerbet, and Nicolab outside the submitted work. JF reports grants and personal fees from Acandis, personal fees from Cerenovus, grants and personal fees from Medtronic, grants and personal fees from Microvention, personal fees from Penumbra, personal fees from Route92, outside the submitted work. IG reports grants from European Union 7th Framework Program during the conduct of the study. VT reports grants from European Union 7th Framework Program during the conduct of the study, and personal fees and non-financial support from Boehringer Ingelheim, Pfizer/BMS, Bayer, Sygnis, Amgen and Allergan, outside the submitted work. RL reports institutional fees from Bayer, Boehringer Ingelheim, Genentech, Ischemiaview, Medtronic and Occlutech outside the submitted work. KM reports grants from European Union 7th Framework Program during the conduct of the study, and personal fees and non-financial support from Boehringer Ingelheim, outside the submitted work. SP reports grants from European Union 7th Framework Program during the conduct of the study. CS reports grants from Novo Nordisk Foundation and personal fees from Bayer, outside the submitted work. CG reports, outside the submitted work, funding from German Research Council (DFG), European Union, Federal Ministry of Education and Research (BMBF), German Statutory Pension Insurance Scheme (RV Nord), National Innovation Fond, Wegener Foundation, Hertie Foundation, and Schilling Foundation, and received, outside the submitted work, personal fees from Abbott, Amgen, Bayer Vital, Bristol-Myers-Squibb, Boehringer Ingelheim, Daiichi-Sankyo, Sanofi Aventis, and Prediction Biosciences. GT reports grants from European Union 7th Framework Program during the conduct of the study and personal fees from Acandis, Boehringer Ingelheim, BMS/Pfizer, Stryker, Daiichi Sankyo, grants and personal fees from Bayer, grants from Corona Foundation, German Innovation Fonds and Else Kroener Fresenius Foundation, outside the submitted work. The remaining authors declare that the research was conducted in the absence of any commercial or financial relationships that could be construed as a potential conflict of interest.
